# Long-Term and Transfer Effects of an Action Control Intervention in Overweight Couples: A Randomized Controlled Trial Using Text Messages

**DOI:** 10.3389/fpsyg.2021.754488

**Published:** 2021-11-24

**Authors:** Corina Berli, Urte Scholz

**Affiliations:** ^1^Applied Social and Health Psychology, Department of Psychology, University of Zurich, Zurich, Switzerland; ^2^University Research Priority Program “Dynamics of Healthy Aging”, Department of Psychology, University of Zurich, Zurich, Switzerland

**Keywords:** randomized controlled trial, text messages, action control, long-term effects, romantic couples, transfer effects, physical activity, accelerometer

## Abstract

Keeping a physically active lifestyle requires consistent self-regulatory effort such as action control (e.g., continuously monitoring and evaluating a behavior in terms of one’s goals). Involving the romantic partner in interventions might be particularly effective in the long run. The present study examined the long-term and transfer effects of an action control intervention in couples using text messaging for promoting target persons’ and partners’ physical activity, anthropometric measures and physical fitness 6 months post baseline. A total of 121 overweight and obese romantic couples, randomly allocated to an intervention (*n* = 60; information + action control text messages) or a control group (*n* = 61; information only) and to participating as target person or partner, completed baseline assessments (T1). 100 couples (82.6%) completed the 6-month follow-up (T3) assessment. Primary outcomes included self-reported moderate-to-vigorous physical activity (MVPA) and objective MVPA and MVPA adherence using triaxial accelerometers across a diary period of 14 days after T3. Secondary outcomes included BMI, waist-to-hip circumference and physical fitness (target persons only) using a submaximal aerobic cycle test. At T3, there were no significant between-group differences between target persons and partners with regard to their objective MVPA, self-reported MVPA, BMI, waist-hip ratio or physical fitness. No significant changes in outcomes were observed from T1 to T3; however, changes in BMI from T1 to T3 between target persons and partners in the intervention group were associated. Overall, the brief 14-days action control intervention was not effective in improving target person’s physical activity, body measures and physical fitness in the long-term. Moreover, no long-term benefits for partners emerged. While brief ecological momentary interventions might be a promising tool for short-term effects, future studies are needed to test features enhancing long-term effectiveness. Associations in romantic partners’ changes suggest that dyadic interventions can be a promising approach, as changes induced in one partner may then transfer over to the other (controlled-trials.com ISRCTN15705531).

## Introduction

Physical inactivity is a major risk factor for the incidence of cardiovascular disease, some types of cancer and type 2 diabetes, and mortality, contributing to over five millions deaths per year globally (WHO, 2020). Despite this, 26% of men and 35% of women in high-income countries are insufficiently physically active (WHO, 2020). Also, prevalence of overweight and obesity have almost tripled during the last 50 years (WHO, 2021). Current guidelines recommend that adults should engage in at least 150 min of moderate-intensity aerobic activity (e.g., 30 min for 5 days a week), or 75 min of vigorous-intensity aerobic activity (WHO, 2020). However, even with best intentions, engaging in regular physical activity in daily life requires tremendous self-regulatory effort (Schwarzer, 2008). Targeting self-regulatory effort in interventions to increase physical activity, particularly in overweight and obese individuals, is thus of high relevance. Additionally, it is crucial to determine whether increases in physical activity can be maintained (i.e., intervention has significant positive effects at least 6 months post baseline, Murray et al., 2017). Long-term adherence to physical activity interventions poses a challenge, and has shown to be below recommended goals (Findorff et al., 2009). This study investigated the long-term and transfer effects across 6 months of an action control intervention in overweight or obese couples using text messaging.

### Using Text Messages to Facilitate Action Control

Action control is an important volitional self-regulatory strategy and refers to continuously monitoring and evaluating a behavior in terms of one’s goals (Sniehotta et al., 2006). It has three subfacets that work jointly in a feedback loop (Sniehotta et al., 2006): (a) being aware of one’s intentions (i.e., *awareness of standards*), (b) keeping track of one’s behavior (i.e., *self-monitoring*) and (c) taking effort to reduce potential discrepancy between the two (i.e., *self-regulatory effort*). Using text messages to facilitate action control could provide an ideal means to reach people in their everyday life and natural context (i.e., ecological momentary intervention, Heron and Smyth, 2010), by reminding of goals, encouraging self-monitoring of behavior and, if necessary, prompting engagement in goal-directed means. This might be particularly relevant as studies have shown that individuals with high adherence to the protocol of physical activity interventions (e.g., attending session, completing homework, or self-monitoring behavior) are also more likely to meet activity recommendations 6 months later than individuals with low adherence (Heesch et al., 2003).

Over the past two decades, there has been a surge in interventions using mobile technology (mHealth) to promote health behavior. Reviews and meta-analyses overall support their effectiveness for changing physical activity, sedentary behavior and weight loss (Fanning et al., 2012; Stephens and Allen, 2013; Yang and Van Stee, 2019), particularly in the short-term (Stephenson et al., 2017). Text messaging is a simple, relatively low cost and efficient technology with far reach (Head et al., 2013; Yang and Van Stee, 2019). 97% of the United States adult population owns a cellphone (Pew Research Center, 2021). Evidence from a meta-analysis shows that text-message interventions have small to medium positive effects, and are particularly effective in the context of physical activity (Head et al., 2013). With a mean follow-up time period of around 12 weeks only (Head et al., 2013), however, studies investigating longer-term follow-ups are scarce. Haapala et al. (2009) could show that overweight individuals participating in a mobile phone weight-loss program with text messages instructing on food intake reductions and providing tailored feedback lost more weight over 12 months compared to controls. More evidence from longer-term follow-ups and objective behavioral measures is highly needed (Stephenson et al., 2017). Also, text message interventions should be grounded in theory to ensure effective intervention methods (Fanning et al., 2012).

### Leveraging the Impact of the Partner

Typically, physical activity interventions focus on the one individual receiving the intervention. However, evidence compellingly shows that health behavior is inextricably intertwined in close relationships (Homish and Leonard, 2008). For example, fluctuations in daily physical activity levels over time co-vary between romantic partners (Berli et al., 2018a). Moreover, findings from a large epidemiological study showed that when one partner changed to a healthier behavior (smoking cessation, physical activity, weight loss), the other partner was more likely to make a positive health behavior change than if their partner continued the unhealthy behavior (Jackson et al., 2015). At the same time, however, having an obese partner, as opposed to a partner with normal weight, increased one’s own risk of becoming obese by 37% (Christakis and Fowler, 2008). To leverage the impact of the partner, there has been a rise in dyadic interventions involving the romantic partner more or less actively in efforts to regulate health behavior (Arden-Close and McGrath, 2017). Involving the partner in the intervention might enhance longer-term success because in sharing their daily life and routines, partners may serve as a continued source of social support, role modeling, feedback, etc. (Richards et al., 2018). To date, couple-based interventions have overall shown promising results in improving physical activity behavior and reduce sedentary behavior (Richards et al., 2018; Carr et al., 2019).

What has been studied less explicitly, however, is whether couple interventions have potential benefits for partners who were not the target of the intervention. Given the high concordance of health behaviors in couples reviewed above, it is not surprising that some studies reported so called ‘transfer effects’ (or ‘ripple effect’) for non-targeted partners (Gorin et al., 2008). For example, Gorin et al. (2008) found that partners of overweight target persons with type 2 diabetes who participated in an intervention to reduce body weight, also showed greater weight reduction 1 year after the intervention compared to partners of target persons in the control condition. Moreover, positive associations between dietary changes in overweight and obese target persons participating in an intervention and changes in their partner’s body weight were found (Schierberl Scherr et al., 2013). No effect on untreated partners’ weight of a lifestyle intervention for pregnant women was found (Hagobian et al., 2019). Other couple intervention studies did not find evidence for increased physical activity in spouses of patients in cardiac rehabilitation (Yates et al., 2015), spouses of prostate cancer survivors (Winters-Stone et al., 2016), and spouses of patients aiming to reduce cholesterol level (King et al., 2014). Knoll et al. (2017) investigated the effects of a dyadic planning intervention, in which target persons were asked to create specific plans with their partner for the target person’s physical activity, compared to an individual planning and control condition. They found no superior effect of the dyadic planning condition on target person’s accelerometer-assessed physical activity over 6 weeks, but an initial increase in partners’ vigorous physical activity 1 week after the intervention. Long-term analyses of the intervention did not reveal beneficial intervention effects across 52 weeks on target person’s moderate-to-vigorous physical activity, but improvements in partners physical fitness (Keller et al., 2020).

### The Present Study

To summarize, interventions using mobile technology such as text messages have the potential to facilitate self-regulation to promote physical activity, however, longer-term effectiveness needs to be further established. Dyadic approaches, involving partners into the intervention, have shown to be promising and might be particularly well-suited for continued effectiveness of physical activity interventions due to the shared environment of couples. Also, there might be benefits for partners that were not the focus of the intervention. So far, evidence on potential transfer effects on participating partners remains inconclusive.

With the present study we aimed to examine the long-term and transfer effects over 6 months of a theory-based action control intervention using text messages to promote daily physical activity in overweight and obese couples (for details see study protocol, Scholz and Berli, 2014). We previously reported on the short term intervention effects for target persons from day-to-day analyses (Berli et al., 2016). We found that the action control intervention effectively enhanced target person’ daily adherence to physical activity recommendations during 14 days of intervention (daily action control text messages) and 14 days of assessment only following the intervention. Exploratory analyses did not reveal further benefit for target persons who received text messages from their partners (dyadic version of the intervention) as compared to from the study staff (i.e., individual version of the intervention).

Specifically, this study investigated the effects of the intervention on *target persons’* physical activity (primary outcome), anthropometric measures including body mass index (BMI) and waist-to-hip-ratio, and physical fitness (secondary outcomes), and *partners’* physical activity and anthropometric measures 6 months post baseline. We hypothesized that target persons and partners of the intervention group would be more successful in improving outcomes than target persons and partners of the control condition (receiving a standard information intervention only). We additionally explored whether changes in these outcomes from baseline to the 6-month follow-up between target persons and their partners were correlated.

## Materials and Methods

### Design and Procedure

This study is part of the single-blind randomized controlled trial ‘A Dyadic Action Control Trial in overweight and obese Couples’ (DYACTIC), aiming to promote daily physical activity in inactive and overweight couples intending to become physically active (see study protocol, Scholz and Berli, 2014). In couples randomized to the intervention group, one partner (randomized to participate as the target person) received an action control text message intervention (delivered either as an individual or a dyadic version of the intervention), in couples randomized to the control group target persons received a standard information intervention only. The project was funded by the Swiss National Science Foundation (PP00P1_133632/1) and approved by the Internal Review Board of the University of Bern, Switzerland (2011-12-36206). The trial was registered at controlled-trials.com (ISRCTN15705531).

Upon completing a short online questionnaire assessing socio-demographic variables and any pre-existing health risks (T0), participating couples were invited to the lab for a baseline assessment (T1). They provided written informed consent, completed a questionnaire, anthropometric measurements (i.e., height, weight, waist and hip) and target persons performed a submaximal aerobic fitness test on a cycle ergometer (for detailed information see Measures section). On the following day, a 28-day diary period started including electronic end-of-day diaries on a study smartphone and accelerometers for all participants. During the first 14 days, the text message intervention took place, followed by 14 days of assessment (end-of-day diaries and accelerometer) without intervention. After this period, approximately 1 month after baseline (T2), couples returned to the lab to return the devices and to complete a follow-up questionnaire and anthropometric measurements. Six months after baseline (T3), they returned to the lab to complete questionnaires, anthropometric measurements, and to perform an aerobic fitness test (target persons only), followed by another 14-day diary period with electronic end-of-day diaries and accelerometers for all participants (assessments only). At the end of this period, they returned the devices via mail. Couples completing T3 were compensated with a total of CHF 200 (= approximately 220 USD).

### Participants and Recruitment

Participants were adult heterosexual couples living in a committed relationship for at least 12 months and cohabiting for at least 6 months. Eligibility criteria were that both partners were overweight or obese (body mass index [BMI] ≥ 25 kg/m^2^), physically insufficiently active (<30 min per day of moderate-to-vigorous physical activity [MVPA]), but intended to engage in the recommended physical activity levels. Participants had to be between 18 and 75 years of age, fluent in German, and be able to receive and read text messages throughout the day. Exclusion criteria were 24 h shift work (to ensure that their partners had the same circadian rhythm), pregnancy and current enrollment in a professional weight loss program. Participants were recruited from the community via advertisements, flyers, and a market research institution. Recruitment took place from March 2012 until October 2013 in Bern, Switzerland.

### Randomization

Randomization was conducted based on a computer-generated allocation sequence that was concealed in a set of sealed, numbered envelopes. In preparing baseline materials, study staff members randomly assigned couples to the intervention or control group, and couple members to being the target person or partner, using restricted randomization in blocks of eight (with two couples being assigned each to the dyadic and individual intervention, and four couples to the control group, with alternating gender for the target person).

### Experimental Groups

#### Intervention Group

The action control intervention had three main components: (1) After completing baseline questionnaires at T1, target persons and their partners received an information leaflet with recommendations on health-enhancing physical activity. At the time of the study recommendations were to engage in at least 30 min of MVPA per day performed in bouts of at least 10 min^1^ (Bundesamt für Sport BASPO [Federal Office of Sports] et al., 2009). (2) Subsequently, target persons in the intervention group were then instructed to set specific behavioral goals to achieve the recommended daily MVPA levels, and write them down on a worksheet. (3) Across the following 14 days intervention period, target persons received one action control text message each week day sent at random times (resulting in a total of 10 messages). Each text message targeted at one of the three subfacets of action control, prompting awareness of standards (e.g., “This message is a small reminder of your intentions to be physically active for 30 min each day”), self-monitoring (e.g., “Which of your intentions in terms of physical activity have you already carried out today?),” self-regulatory effort (e.g., “If you haven‘t achieved your goal of 30 min physical activity today, there will certainly still be a good opportunity for it.”). For an overview of all messages see Scholz and Berli (2014). Order of the text messages and time of the day sent was equivalent for all participants. The following behavior change techniques (BCTs; Michie et al., 2013) were addressed in the intervention: information about health consequences, goal setting, self-monitoring of behavior, discrepancy between current behavior and goal standard, and self-monitoring of behavior. The intervention was administered in two versions:

##### Individual action control

Target persons set behavioral intentions individually, and action control text messages were sent from the study staff via an automated system. Partners also received text messages at the same time as target persons, but with a reminder to fill in the end-of-day diary.

##### Dyadic action control

With the assistance of partners, target persons set behavioral intentions to increase the target person’s physical activity to the recommended level. Partners were instructed to send the preset action control text messages with the exact same content but personalized (e.g., dialect, greetings) to the target persons. Partners received a reminder text message from the study staff prompting to send the appropriate action control text message (saved as draft on their study smartphone) to the target person within the next hour, and to fill in the end-of-day diary. Target persons were not informed about what instructions were given to their partner, and were instructed not to discuss them with their partner.

#### Control Group

Target persons and their partners randomized to the control group received the same information leaflet as participants of the intervention group. However, target persons were not asked to set specific behavioral goals. They received text messages at the same time as target persons in the intervention group, but with a reminder to fill in the end-of-day diary.

### Primary Outcome Measures

As primary outcomes, we used self-report and objective measures of moderate-to-vigorous physical activity (MVPA).

#### Self-Reported Moderate-to-Vigorous-Physical Activity (MVPA) per Day

At T1, T2, and T3, target persons and partners filled in a 7-day recall questionnaire on actual physical activity covering activities in the domains of home and garden, transportation, and leisure and sports. The questionnaire was adapted from a physical activity frequency questionnaire (Bernstein et al., 1998) which has been validated in Switzerland (Maeder et al., 2006). A copy of the questionnaire used can be found in the Supplementary Material A. They were asked to indicate for each activity on how many days of the previous 7 days (frequency) and on average how many minutes per day (duration per occasion) they engaged in this particular activity. Moreover, they could list up to three additional activities. All activities were assigned their respective metabolic equivalent [MET] intensity level based on the compendium of physical activities (Ainsworth et al., 2011). Minutes spent in MVPA per day were summed for all activities of at least moderate intensity (≥3.0 METs) in accordance with physical activity recommendations, resulting in a total of 27 items.

#### Objective Physical Activity per Day

Across 14 days following the 6 months follow-up (T3), objective daily physical activity was assessed from target persons and partners with a triaxial accelerometer monitoring device (GT3X+, ActiGraph, Pensacola, FL, United States). Participants were instructed to wear the monitor at the hip on the side of the dominant hand from the moment they got up in the morning until they went to bed at night, and to remove it only for showering or water-based activities lasting more than 30 min. Data was processed in ActiLife 6 software (for details on data processing please see Berli et al., 2016). Only days with at least 10 h of valid wear time were included in the analyses, with non-wear time filtered based on an algorithm of ≥90 min of consecutive zeros in vector magnitude (Choi et al., 2011). From the 99 couples completing the diary period at T3, accelerometer data was missing from two couples due to technical issues. This resulted in *N* = 97 target persons with 1219 (89.8%) available valid days, and *N* = 97 partners with 1209 (89.0%) available valid days. In line with primary outcomes assessed during the intervention and follow-up period after T1 (see Berli et al., 2016), we computed two scores: (a) total minutes of *objective MVPA per day*, calculated based on the threshold of ≥2,690 cpm in vector magnitude to identify activity with at least moderate intensity (Sasaki et al., 2011), and (b) *objective MVPA adherence*, based on recommendations for health-enhancing physical activity in Switzerland at the time of the study (Bundesamt für Sport BASPO [Federal Office of Sports] et al., 2009), using the total minutes of moderate physical activity per day that was performed in bouts of at least 10 min. Days with 30 or more minutes of MVPA performed in bouts of at least 10 min were coded as 1 (adherent days), days with less than 30 min were coded as 0 (non-adherent days). For both outcomes we created a mean score per person across the 14 days to represent an average objective daily physical activity per person.

### Secondary Outcome Measures

As secondary outcomes of the intervention, we investigated two anthropometric measures that have been shown to be associated with increased risk of morbidity and mortality (WHO, 2008). Body mass index (BMI) is a simple and globally accepted indicator for measuring obesity in general, without providing information about body fat distribution. Waist-to-hip ratio (WHR) is a screening tool to determine abdominal overweight and obesity (Tutunchi et al., 2020). Moreover, to assess changes in physical fitness as a strong predictor of physiological health outcomes such as cardiovascular disease, cancer, and mortality (Blair et al., 2001), we included an aerobic measure of physical fitness.

#### Body-Mass Index

At T1, T2, and T3, body weight and height measurements of target persons and partners were taken by the study staff, and BMI was calculated (kg/m^2^).

#### Waist-to-Hip Ratio

At T1, T2, and T3, waist and hip circumference of target persons and partners were measured by the study staff using a stretch-resistant tape parallel to the floor. Waist circumference was measured approximate midpoint between the lower margin of the last palpable rib and the top of the iliac crest; hip circumference was measured around the widest portion of the buttocks (WHO, 2008). Waist-to-hip ratio was then calculated (waist/hip).

#### Physical Fitness

At T1 and T3 and only in target persons^2^, objective physical fitness was measured with a submaximal aerobic exercise test on a cycle ergometer, which is most appropriate for use in an overweight and obese population and presents a less stressful alternative to maximal aerobic exercise testing as the gold standard (Wallman and Campbell, 2007). We used the Aerobic Power Index (API; Telford et al., 1989), a highly reliable protocol in sedentary and obese populations (Wallman et al., 2003; Wallman and Campbell, 2007). This exercise protocol consists of pedaling at 25 watts (W) for 1 min, increasing by 25 W every subsequent minute until the target person reaches his or her target heart rate. Target heart rate is 75% of the predicted maximal heart rate. We used the following formula accurately predict maximal heart rate in overweight and obese adults: 208 – age × 0.7 (Franckowiak et al., 2011). The test ends at the end of the minute that the target heart rate has been achieved. The power output (W) at the target heart rate is determined through interpolation. This result is then divided by the participant’s body weight (in kg), resulting in an aerobic index in watt per kilogram body weight (W kg^–1^).

Before the start of the test, target persons were given detailed information about the procedure, conditions for participation were checked (i.e., no preexisting health conditions, no intake of beta blocker), and they signed a consent statement. Then, target persons were asked to put on a heart rate monitor around the chest and were seated on a cycle ergometer (Siemens EM840) in an upright position so that their knees were slightly flexed with their foot placed on the pedal at the lowest point. They were instructed to keep the pedaling frequency as consistent as possible at a minimum of 60 cycles per minute (visible on a display). Resting heart rate was assessed, and then the test started at 25 W. At the end of each minute, the study staff registered power output and ratings of perceived exertion. In case participants experienced fatigue, dizziness, palpitation, pain, or did not want to continue for any other reason, the test could be ended early anytime, and the highest output achieved was used to calculate the score.

Data from the fitness test was available from 92 (76%) and 72 (72%) target persons at T1 and T3, respectively. Missings were mainly due to the intake of beta-blocker (15% and 15%), followed by health issues (2% and 8%), technical problems (2% and 6%) at T1 and T3, respectively.

### Data Analysis

Data were analyzed for target persons and partners separately, using IBM SPSS 26. Preliminary analyses included dropout analyses, randomization and manipulation checks that were performed using χ^2^ and two-tailed *t*-tests for independent groups. To investigate differences between the intervention and control group in levels of target persons’ and partners’ self-reported and objective physical activity, BMI, waist-to-hip ratio and physical fitness (target persons only) 6 months post baseline (T3), we performed two-tailed *t*-tests for independent groups. As effect size measure for significant group differences, we reported Cohen’s *d* with 0.20, 0.50, and 0.80 indicating small, medium and large effects, respectively (Cohen, 1988). To test for changes over time and differential changes across groups, we performed a two-way mixed ANOVA for all outcomes but objective physical activity with time as a within-subject factor and intervention group as a between-subject factor, using outcome data from baseline (T1), 1-month follow-up (T2), and 6-months follow-up (T3). A significant effect of time indicates that there are significant changes in outcomes over time overall (across both groups). A significant interaction effect of time and group indicates that changes were different for the intervention and control group. As effect size measure, we reported the partial eta square with $\eta_{\text{p}}^{2}$ < 0.01 for small, $\eta_{\text{p}}^{2}$ < 0.06 for medium, and $\eta_{\text{p}}^{2}$ < 0.14 for large effect (Cohen, 1988). Lastly, to explore whether changes in outcomes over time were associated within couples, we calculated bivariate correlations between changes in outcomes from T1 to T3 in target persons and partners.

## Results

### Sample and Dropout

**FIGURE 1:**
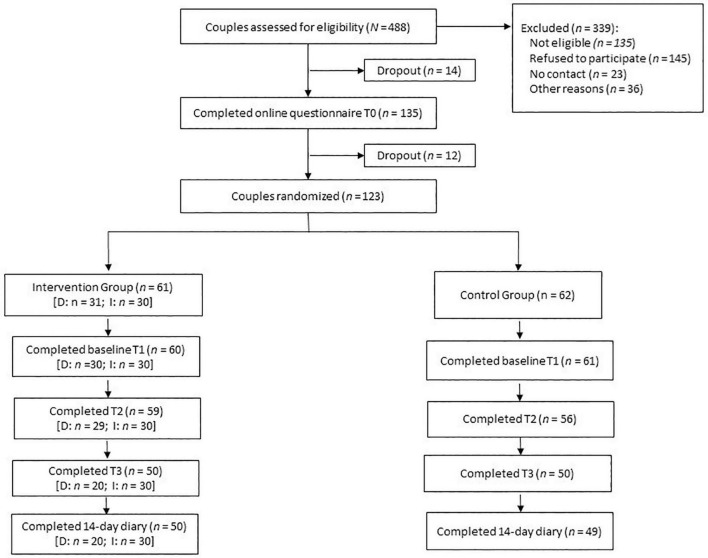
Flow chart of participating couples. D, dyadic version of intervention; I, individual version of intervention.

Of the 121 couples (*n* = 61 in the control group, *n* = 60 in the intervention group) who completed baseline assessments, 115 couples (95.0%) participated at T2 and 100 couples (82.6%) participated at T3 (see flow chart in Figure 1). Mean age was 46.13 (13.62) years for target persons (51.2% female), and 46.07 (13.70) years for partners. Target persons had a mean BMI of 31.00 (5.58), partners had a mean BMI of 31.22 (4.25). Most target persons and partners were employed (65.3% and 72.7%, respectively). According to reports of target persons, most were married (69.4%), had children (57%), and had been living in their relationship for 18.79 (14.33) years. Table 1 displays baseline characteristics for intervention and control group separately.

**TABLE 1:** Sample characteristics at baseline (T1).

	Target persons			Partners		
T0-indicators	IG (*n* = 60)	CG (*n* = 61)	χ^2^	IG (*n* = 60)	CG (*n* = 61)	χ^2^
Female (%)	51.7	50.8	0.01	48.3	49.2	0.01
Married (%)	76.7	62.3	2.94	76.7	62.3	2.94
Having children (%)	61.7	52.5	1.05	61.7	52.5	1.05
Employed (%)	65.0	65.6	0.004	70.0	75.4	0.45

			***t***			***t***

Age in years	48.33(13.13)	43.97(13.87)	−1.78	47.68(13.54)	44.49(13.78)	−1.29
Body mass index (kg/m^2^)	31.71(6.48)	30.26(4.48)	−1.43	31.93(4.53)	30.52(3.87)	−1.84
Relationship duration (years)	19.56(14.27)	18.03(14.48)	−0.59	19.60(14.21)	17.90(14.26)	−0.66
Cohabitation duration (years)	18.17(14.11)	15.95(14.56)	−0.85	18.18(14.58)	15.86(14.60)	−0.89
Baseline intentions	4.98(0.61)	4.70(0.72)	−2.30*	4.78(0.64)	4.88(0.70)	0.86
Baseline action control	3.18(1.24)	2.86(1.10)	−1.49	3.26(1.14)	2.86(0.96)	−2.10*

*IG, intervention group; CG, control group.*

*p < 0.05.

Participating couples who dropped out before T3 were similar to those who completed T3 in terms of target persons’ and partners’ baseline characteristics such as gender, age, relationship length, being employed, baseline intentions, and action control (*p* > 0.05). Neither did they differ in terms of key outcome variables at the baseline assessment (T1), including self-reported physical activity, BMI and waist-to-hip ratio, physical fitness or group assignment (*p* > 0.05). Thus, it can be assumed that the sample with data at T3 is representative of the overall sample.

### Randomization Check

Randomization check for target persons has been previously reported (Berli et al., 2016). As can be seen in Table 1, target persons and partners in the intervention and control group did not significantly differ in most baseline characteristics, including age, relationship length, duration of cohabitation, BMI (*p* > 0.05) (see Table 1). However, in the intervention group target persons reported higher baseline physical activity intention [*M*_*IC*_ = 4.98(0.61) vs. *M*_*CG*_ = 4.70(0.72); *t*(199) = −2.30, *p* = 0.023, *d* = 0.42], and partners reported higher action control [*M*_*IC*_ = 3.26(1.14) vs. *M*_*CG*_ = 2.86(0.96); *t*(199) = −2.10, *p* = 0.038, *d* = 0.38]. In sensitivity analyses, we checked whether including these variables in respective target persons and partner models would change the pattern of results. As this was not the case, we reported the models without covariates below.

### Manipulation Check

Increases in action control from baseline levels (T1) to the 1-month follow-up (T2) were significantly higher for target persons in the intervention group (*M* = 1.10, *SD* = 1.26) compared to target persons in the control group [*M* = 0.58, *SD* = 1.17, *t*(113) = −2.27, *p* < 0.05, *d* = 0.43]. There were also increases in action control for partners in the intervention group (*M* = 0.74, *SD* = 1.26), but not significantly different from increases for partners in the control group [*M* = 0.57, *SD* = 0.97, *t*(113) = −0.81, *p* = 0.420, *d* = 0.15]. At the 6 months follow-up (T3), reports of action control from target persons and partners did not significantly differ across groups.

### Intervention Fidelity

As reported previously (Berli et al., 2016), seven target persons (24.1%) received less than half of the messages in the intended manner during the intervention period, and were considered as low fidelity participants. Excluding these seven persons from the analyses did not change the pattern of results.

### Descriptives

Descriptive statistics for outcome measures of target persons and partners in the intervention and control group at baseline (T1), the 1-month follow-up (T2) and the 6-month follow-up (T3) are displayed in Table 2. At T3, target persons and partners from both groups reported on average to engage in 144 and 159 min of MVPA per day, respectively. Objective assessments via accelerometer at T3 showed that on average, target persons and partners were active with at least moderate intensity for 48.5 and 48.0 min per day, and adhered to recommended MVPA levels on 23% and 22% percent of the days, respectively. At T3, BMI was on average 31.3 (43.9% with BMI < 30) and 31.9 (33.3% with BMI < 30) for target persons and partners, respectively. Waist-to-hip ratio was on average 0.92 and 0.91 for target persons and partners, respectively, indicating substantially increased risk of metabolic complications (WHO, 2008). Target persons achieved an average workload of 1.5 (per kg weight), reflecting a fair to average aerobic fitness score.

**TABLE 2:** Descriptive statistics of primary and secondary outcomes for target persons and partners at baseline (T1), 1-month (T2) and 6-months (T3) follow-up.

		Target persons			Partners		
		Intervention (*n* = 60)	Control (*n* = 61)		Intervention (*n* = 60)	Control (*n* = 61)	
				
		*M (SD)*	*M (SD)*	*t*	*M (SD)*	*M (SD)*	*t*
Self-reported MVPA (min/day)	T1	157.06 (198.65)	141.97 (146.76)	–0.48	185.21 (183.27)	131.86 (173.75)	–1.64
	T2	172.43 (175.60)	170.83 (176.75)	–0.05	173.71 (180.69)	137.59 (153.10)	–1.15
	T3	142.17 (126.75)	146.02 (147.26)	0.14	168.72 (167.04)	148.99 (177.82)	–0.57
Objective MVPA (min/day)	T3	46.73 (27.16)	50.22 (23.90)	0.67	45.81 (22.05)	50.38 (24.62)	0.96
Objective MVPA adherence	T3	0.24 (0.25)	0.22 (0.22)	–0.43	0.20 (0.19)	0.24 (0.21)	0.95
BMI	T1	31.96 (5.54)	30.63 (4.02)	–1.51	32.60 (4.71)	31.20 (3.82)	–1.79
	T2	31.92 (5.54)	31.05 (4.77)	–0.88	32.27 (5.00)	30.97 (3.64)	–1.54
	T3	31.88 (5.27)	30.76 (4.78)	–1.10	32.35 (4.43)	31.47 (4.06)	–1.03
Waist-to-hip ratio	T1	0.92 (0.07)	0.91 (0.09)	–0.37	0.92 (0.09)	0.92 (0.09)	0.31
	T2	0.91 (0.08)	0.92 (0.08)	1.03	0.91 (0.09)	0.91 (0.09)	–0.30
	T3	0.92 (0.07)	0.92 (0.09)	0.47	0.92 (0.09)	0.91 (0.08)	–0.11
Physical fitness (Wkg^–1^)	T1	1.40 (0.42)	1.50 (0.39)	1.20	–	–	–
	T3	1.44 (0.39)	1.60(0.45)	1.59	–	–	–

*T1: N = 121; T2: N = 115; T3: N = 100; M, mean; SD, standard deviation. For all t’s p > 0.05.*

### Long-Term Intervention Effects on Target Persons

As can be seen in Table 2, there were no significant differences between target persons in the intervention and control group in terms of T2 levels of *self-reported MVPA* (*p* = 0.961), *BMI* (*p* = 0.379) or *waist-to-hip ratio* (*p* = 0.305), and T3 levels of *self-reported MVPA* (*p* = 0.889), *objective MVPA* (*p* = 0.503), *objective MVPA adherence* (*p* = 0.671), *BMI* (*p* = 0.273), *waist-to-hip ratio* (*p* = 0.640), or *physical fitness* (*p* = 0.117).

**FIGURE 2:**
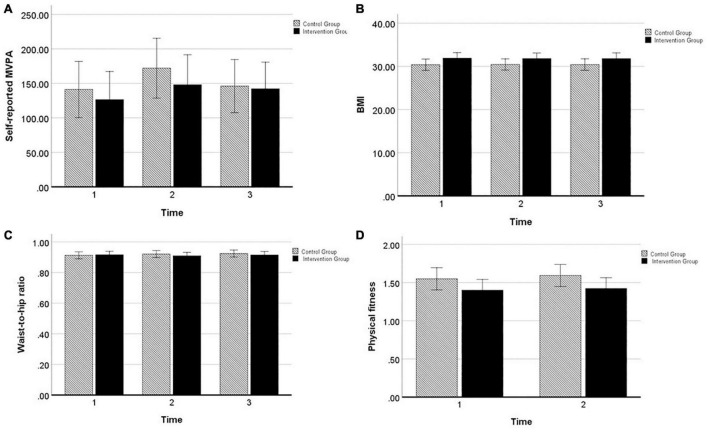
Profile plots from two-way mixed ANOVA’s of estimated marginal means of target persons’ **(A)** self-reported MVPA, **(B)** BMI, **(C)** waist-to-hip ratio, and **(D)** physical fitness, displayed for intervention group (black) and control group (black–white striped) with 95% confidence interval error bar.

Figure 2 displays results from the two-way mixed ANOVAs (with time as within-subject factor and group as between-subject factor). For target persons’ *self-reported MVPA*, there was no significant effect of group [*F*(1,98) = 0.36, *p* = 0.553, $\eta_{\text{p}}^{2}$ = 0.004], time [*F*(2,196) = 1.61, *p* = 0.202, $\eta_{\text{p}}^{2}$ = 0.02], or time by group [*F*(2,196) = 0.23, *p* = 0.792, $\eta_{\text{p}}^{2}$ = 0.002]. For target persons’ *BMI*^3^ there was no significant effect of group [*F*(1,93) = 2.40, *p* = 0.125, $\eta_{\text{p}}^{2}$ = 0.03], time [*F*(1.5,143.4) = 0.13, *p* = 0.969, $\eta_{\text{p}}^{2}$ < 0.001], or time by group [*F*(1.5,143.4) = 0.21, *p* = 0.754, $\eta_{\text{p}}^{2}$ = 0.002]. For target persons’ *waist-to-hip ratio* there was no significant effect of group [*F*(1,95) = 0.15 *p* = 0.695, $\eta_{\text{p}}^{2}$ = 0.002], time [*F*(2,190) = 0.51, *p* = 0.600, $\eta_{\text{p}}^{2}$ = 0.01], or time by group [*F*(2,190) = 1.20, *p* = 0.305, $\eta_{\text{p}}^{2}$ = 0.01]. For target persons’ *physical fitness* there was no significant effect of group [*F*(1,66) = 2.59, *p* = 0.113, $\eta_{\text{p}}^{2}$ = 0.04], time [*F*(1,66) = 2.56, *p* = 0.115, $\eta_{\text{p}}^{2}$ = 0.04], or time by group [*F*(1,66) = 0.25, *p* = 0.618, $\eta_{\text{p}}^{2}$ = 0.004]. For all outcomes, effects were small in size and non-significant, indicating that there were no changes from baseline (T1) to the 6 months follow-up (T3) and no differences between the intervention and control group in changes over time.

Overall, these results disconfirm our hypotheses that target persons of the intervention group would be more successful than target persons of the control group in improving their physical activity and physical fitness, and reducing anthropometric measures until 6 months post-intervention

### Long-Term Intervention Effects on Partners

As displayed in Table 2, there were no significant differences between partners in the intervention and control group in terms of T2 levels of *self-reported MVPA* (*p* = 0.251), *BMI* (*p* = 0.126) or *waist-to-hip ratio* (*p* = 0.762), and T3 levels of *self-reported MVPA* (*p* = 0.569), *objective MVPA* (*p* = 0.338), *objective MVPA adherence* (*p* = 0.347), *BMI* (*p* = 0.307), or *waist-to-hip ratio* (*p* = 0.910).

**FIGURE 3:**
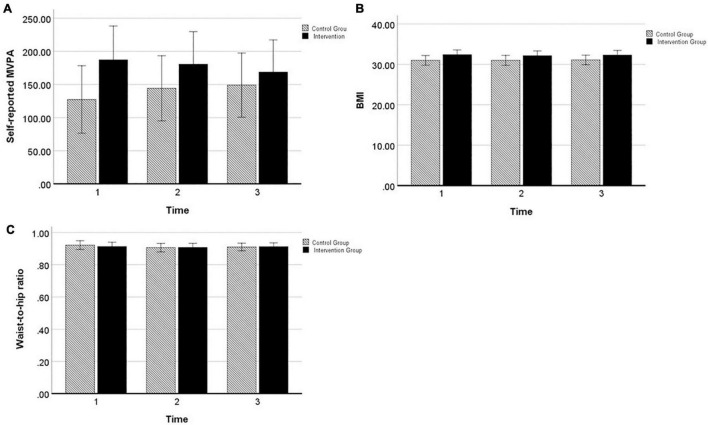
Profile plots from two-way mixed ANOVA’s of estimated marginal means of partners’ **(A)** self-reported MVPA, **(B)** BMI, and **(C)** waist-to-hip ratio, displayed for intervention group (black) and control group (black–white striped) with 95% confidence interval error bar.

Figure 3 displays results from the two-way mixed ANOVAs (with time as within-subject factor and group as between-subject factor). For partners’ *self-reported MVPA*, there was no significant effect of group [*F*(1,98) = 1.71, *p* = 0.194, $\eta_{\text{p}}^{2}$ = 0.02], time [*F*(2,196) = 0.05, *p* = 0.952, $\eta_{\text{p}}^{2}$ = 0.001], or time by group [*F*(2,196) = 7.35, *p* = 0.481, $\eta_{\text{p}}^{2}$ = 0.01]. For partners’ *BMI*^4^, there was no significant effect of group [*F*(1,93) = 2.21, *p* = 0.140, $\eta_{\text{p}}^{2}$ = 0.02], time [*F*(1.45,134.85) = 0.76, *p* = 0.432, $\eta_{\text{p}}^{2}$ = 0.01], or time by group [*F*(1.45,134.85) = 0.67, *p* = 0.466, $\eta_{\text{p}}^{2}$ = 0.01]. For partners’ *waist-to-hip ratio*, there was no significant effect of group [*F*(1,94) = 0.01, *p* = 0.915, $\eta_{\text{p}}^{2}$ < 0.01], time [*F*(2,188) = 1.50, *p* = 0.226, $\eta_{\text{p}}^{2}$ = 0.02], or time by group [*F*(2,188) = 0.46, *p* = 0.634, $\eta_{\text{p}}^{2}$ = 0.01]. For all outcomes, effects were small in size and non-significant, indicating that there were no changes from baseline (T1) to the 6 months follow-up (T3) and no differences between the intervention and control group in changes over time.

Overall, these results disconfirm our hypotheses that partners of the intervention group would be more successful than partners of the control group in improving their physical activity, and reducing anthropometric measures until 6 months post-intervention.

### Correlated Changes in Target Persons and Partners

A significant positive correlation emerged between changes from T1 to T3 in target persons’ and partners’ BMI [*r*(96) = 0.31, *p* < 0.01]. This indicates that target persons whose partners showed a greater decrease in BMI from T1 to T3 were also more likely to show a greater decrease in BMI^5^. No significant correlations between target persons’ and partners’ changes emerged for self-reported MVPA per day [*r*(100) = 0.04, *p* = 0.671] and waist-to-hip ratio [*r*(98) = −0.03, *p* = 0.747]. Further, target persons’ and partners’ levels of objective MVPA [*r*(97) = 0.42, *p* < 0.001] and objective MVPA adherence [*r*(97) = 0.50, *p* < 0.001] at T3 were significantly associated.

### Exploratory Analysis

We also exploratorily tested for differences between the dyadic and individual version of the action control intervention. Only in partners, a significant difference in *objective MVPA adherence* at T3 emerged: Partners in the dyadic intervention group reported a higher average probability to achieve physical activity recommendations (*M* = 0.26, *SD* = 0.22) than partners in the individual action control condition (*M* = 0.16, *SD* = 0.16) [*t*(47) = −1.72, *p* = 0.093, *d* = 0.54]. Otherwise, no significant differences emerged between target persons and partners of the two versions of the intervention. Detailed results can be found in Supplementary Material B (Table S1).

## Discussion

Identifying interventions that effectively increase and maintain physical activity levels in everyday life are essential to tackle the pressing public health issue of physical inactivity and obesity around the globe. This study reports on the long term effects across 6 months of an action control intervention in overweight and obese couples using text messages. Because effects of couple interventions might be particularly likely to transfer to non-targeted partners, we investigated the intervention effects on both, target persons and partners. We used a mix of self-reported and accelerometer-assessed physical activity measures, as well as objective anthropometric and physical fitness measures.

Previously reported findings on the intervention effects during and immediately after the intervention showed that target persons in the intervention group had a higher probability of adhering to the recommended daily MVPA levels (on average on 34% of the days) during the 14 days of intervention and the 14 days following the intervention than target persons in the control group (on average on 22% of the days). No difference was found for the total minutes of daily MVPA (Berli et al., 2016, 2018b). Contrary to our hypotheses, there were no differences 6 months post baseline (T3) between target persons and partners in the intervention and control group in terms of their level of self-reported and accelerometer-assessed physical activity, BMI, waist-to-hip ratio, and physical fitness (target persons only). Further, small and non-significant changes in outcomes assessed repeatedly (except objective physical activity) emerged over time in both groups.

These results indicate that the immediate effect of the intervention in enhancing target persons’ adherence to MVPA levels, which was sustained across 2 weeks after the text messages ended, was not maintained across the 6-month period. Because no further assessments were taken between 1 month and 6 months after baseline, it, however, remains unclear when intervention effects on MVPA adherence faded out. Overall, this finding is in line with results from a meta-analyses on mobile phone interventions for physical activity, demonstrating that effect size were particularly pronounced in the short-term and lessened over time, perhaps due to the “novelty” of the technology wearing off (Stephenson et al., 2017). A study by Wang et al. (2015) even found that automatic daily text messages with simple physical activity prompts increased physical activity levels only for a period of 1 week. What would make text message intervention more effective in the long run? Fourteen days of intervention with ten text messages overall might have been too short to establish physical activity habits. Moreover, text messages have found to be more effective when tailored to the individual (Head et al., 2013). The present action control intervention might thus have shown greater effects when tailored for example to the individual goals, and preferences of recipients in terms of number of texts, etc. Further, combining action control with other self-regulation strategies such as action and coping planning might be more effective to stimulate long term change, as demonstrated in the context of cardiac rehabilitation (e.g., Scholz and Sniehotta, 2006). Also, self-efficacy might facilitate persistent engagement in recommended physical activity levels, particularly in times when mastery experience, an important source of self-efficacy, is low (Bandura, 1977). Another explanation for the null-findings regarding long-term effectiveness may be the lack of booster sessions. The specific goals that the target persons set may not have been up-to-date anymore and had need revisions for participants to be committed to achieving these goals. Also, the salience of the recommendations for minimum activity may have faded over time. Yet, even interventions that include booster sessions are not always successful (Keller et al., 2020). Given that the effect on behavioral target of the intervention was not sustained over time, and results from self-reported physical activity did not indicate much change in the overall amount of MVPA, null findings for secondary outcomes are rather less surprising.

Results also suggest that there were no transfer effects on participating partners. Partners thus did not benefit long term from the action control intervention the target persons received. While this contrasts with some findings from weight loss interventions (Gorin et al., 2008), it echoes findings from several couple interventions in the context of physical activity (Yates et al., 2015; Winters-Stone et al., 2016). The mechanisms behind potential transfer effects still remain unclear. One assumption is that partners mimic health behaviors of the spouses who receive the intervention due to the shared home environment (Hagobian et al., 2019). If we assume a mechanism that drives transfer effects via change in target persons (e.g., role modeling, joint behavioral effort, etc.), there needs to be a change in target persons for transfer effects to emerge. However, there might also be mechanisms independent of target persons. For example, the intervention might stimulate partners’ self-regulation (goal setting, goal salience, self-efficacy), or support (providing of support, reciprocated support) that could directly impact their own behavior (Lüscher et al., 2018; Berli et al., 2021). A dyadic planning intervention (Keller et al., 2020) also showed that distinct effects on target persons and partners are possible. One important feature determining the presence of transfer effects might be how actively partners are involved. Previous research has shown that interventions in the context of physical activity in which the primary focus of the intervention was on the dyad instead of an identified target person might be more effective (Richards et al., 2018). In an additional exploratory analysis (see Supplementary Material B), we did not find differences in long term effects between target persons and partners participating in a dyadic version (active involvement in action control) or individual version (no active involvement in action control) of the action control intervention, except for a marginally higher objective MVPA adherence in partners. This finding hints at the possibility that partners might benefit particularly when being more actively involved, however, due to the small sample size and limited power, such comparisons need to be interpreted with caution.

Moreover, results indicate that in target persons and partners of both groups, on average, self-reported physical activity, BMI, waist-to-hip ratio and physical fitness remained fairly stable over time. This is in contrast with planning interventions which resulted in overall long-term improvements for objective physical activity (Keller et al., 2020) and weight (Knäuper et al., 2018), independent of conditions. One explanation might be that the present sample of inactive, and overweight or obese couples, often with a history of many failed attempts, generally encounters more barriers in changing their physical activity. Change may also have been quite heterogeneous within groups with some participants improving in outcomes and other participants declining in outcomes over time. In line with this assumption, we did find that change from baseline to the 6-month follow-up had a large range. Moreover, our exploratory analysis showed some evidence for concordant change in couple members, in that change in BMI from baseline to the 6-month follow-up was positively associated between target persons and partners. Also, levels of objective physical activity outcomes were positively correlated between target persons and partners, in line with previous findings on health behavior concordance in couples (Wilson, 2002; Homish and Leonard, 2008). The results unfortunately do not shed light on the specific mechanisms that drive concordance in couples, e.g., whether change in one partner drives change in the other or whether change is simultaneous at the level of the dyad, possibly due to dyadic process such as setting joint goals, or planning collaboratively (e.g., Prestwich et al., 2005). Future research should use a micro-time perspective using for example measurement bursts of intensive longitudinal assessments to better examine the dynamics of such processes, or use open-end questionnaires.

### Strengths and Limitations

This study’s strengths are its theory-based and pre-registered intervention design with text messages delivered to people in their everyday lives, the use of objective measures of physical activity and health-related secondary outcomes, and the dyadic assessments of outcomes in both target persons and partners of the participating dyad across a period of 6 months. However, some limitations need to be noted as well. First, because there was no pre-intervention baseline assessment of accelerometer-based activity, it was not possible to test for changes in objective physical activity over time. Self-report was available from all assessments, but is often overestimated compared to objectively assessed physical activity (Prince et al., 2008). Second, as there were no further assessments between the 1- and 6-month follow-up, it is not possible to elucidate when the immediate intervention effect on objective MVPA adherence faded out. Third, enrolling and randomizing romantic couples into the study, did not allow to explicitly test for the effect of partners participating together with the target person in the study, receiving a standard information intervention and completing assessments. It might be that such “mere presence” of the partner sets off important mechanisms in the control group as well, obscuring potential group differences (Richards et al., 2018). This would however have required an additional experimental group, which given the already strict inclusion criteria in a specific population sample would not have been feasible.

## Implications and Conclusion

The added value of the study consists of providing evidence on the effectiveness of a theory-based intervention in couples’ everyday life and the potential benefits for both partners’ long-term behavior change. The present findings thus have important implications for future research and health promotion applications. Results of the present study add to the evidence that brief theory-based text message interventions might be less successful for effectively changing physical activity in daily life over longer time periods, despite their potential due to simple and low cost technology, and scalability (Marcolino et al., 2018). Results further demonstrate the difficulty to change rather slow-changing health-related indicators such as BMI or physical fitness. Future research should more systematically investigate the conditions under which text message interventions may unfold benefits over longer time periods, including the use of booster sessions. Also, alternative approaches need to be tested, such as using text messages in combination with apps, which was found to be more effective than using only one of these technology types (Yang and Van Stee, 2019). Because apps can be used beyond the intervention period and integrated in personal daily life, this might enhance long-term effectiveness. Further, using gamification (i.e., integrating game elements in non-game contexts, Johnson et al., 2016) in apps might be an effective approach to promote long-term behavior change. Gamification is assumed to sustain engagement with personal applications and to enhance positive user experience, and has shown to have particularly positive effects on health behaviors (Johnson et al., 2016). Positive effects of gamification on physical activity has been shown in a sample of obese veterans, but only over shorter-term periods (Agarwal et al., 2021).

Further, the present study could not demonstrate long-term benefits for participating partners. Given the findings on behavioral concordance between target persons and partners, however, interventions targeting couples are still promising. If successful, couple intervention have the potential to be an efficient and cost-effective approach to initiate and maintain health behavior change (Richards et al., 2018). To move the field forward, there is a strong need for future work to adequately address the heterogeneity of couple interventions. This also involves to more systematically identify and differentiate between varying degrees of partner involvement and specific dyadic techniques employed (Scholz et al., 2020). Lastly, interventions usually do not work equally well for everyone. Given the variation in change across participants, future work should more systematically consider moderating variables. Relationship quality might for example be an important baseline feature of couple interventions (Keller et al., 2020), facilitating effective dyadic processes to unfold.

To conclude, no long term effects of an action control intervention in couples using text messages were found for target persons and partners. Evidence for behavioral concordance between target persons and partners, however, suggests that if change can be induced in one partner, it may transfer over to the other. Future research should investigate possibilities to use text messages more effectively to maintain initial intervention effects; and how to include partners effectively to maximize the impact of interventions with couples.

## Data Availability Statement

The raw data supporting the conclusions of this article will be made available by the authors, without undue reservation to any qualified researcher.

## Ethics Statement

The studies involving human participants were reviewed and approved by the Ethics Commission of the Faculty of Human Sciences of the University of Bern, Switzerland (2011-12-36206). The patients/participants provided their written informed consent to participate in this study.

## Author Contributions

US acquisited funding, designed the empirical study, and critically reviewed and edited the manuscript. CB supervised data collection, led the project administration, conceptualized the aims of the present manuscript, analyzed the data, and wrote the original draft of the manuscript. Both authors approved the final submitted version of the manuscript.

## Conflict of Interest

The authors declare that the research was conducted in the absence of any commercial or financial relationships that could be construed as a potential conflict of interest.

## Publisher’s Note

All claims expressed in this article are solely those of the authors and do not necessarily represent those of their affiliated organizations, or those of the publisher, the editors and the reviewers. Any product that may be evaluated in this article, or claim that may be made by its manufacturer, is not guaranteed or endorsed by the publisher.

## Footnotes

1 In 2013 these guidelines were updated to meet the global standards of the WHO and CDC specifying a minimum weekly amount.

2 Physical fitness was only assessed in target persons to keep the burden of the laboratory assessment in terms of time and strain reasonable, given that the focus of the intervention was on target persons.

3 Because the sphericity assumption was not met for target persons’ BMI, we reported the statistics from the Greenhouse–Geisser correction.

4 Because the sphericity assumption was not met for partners’ BMI, we reported the statistics from the Greenhouse–Geisser correction.

5 47.4% of target persons and 43.9% of partners showed a decrease in BMI from T1 to T3.

## References

[B1] Agarwal A. K. Waddell K. J. Small D. S. Evans C. Harrington T. O. Djaraher R. (2021). Effect of gamification with and without financial incentives to increase physical activity among veterans classified as having obesity or overweight: a randomized clinical trial. *JAMA Netw. Open* 4:e2116256. 10.1001/jamanetworkopen.2021.16256 PMC8271358 34241628

[B2] Ainsworth B. E. Haskell W. L. Herrmann S. D. Meckes N. Bassett D. R. Tudor-Locke C. (2011). Compendium of physical activities: a second update of codes and MET values. *Med. Sci. Sports Exerc.* 43 1575–1581. 10.1249/MSS.0b013e31821ece12 21681120

[B3] Arden-Close E. McGrath N. (2017). Health behaviour change interventions for couples: a systematic review. *Br. J. Health Psychol.* 22 215–237. 10.1111/bjhp.12227 28150410 PMC5408388

[B4] Bandura A. (1977). Self-efficacy: toward a unifying theory of behavioral change. *Psychol. Rev.* 84 191–215. 10.1037//0033-295x.84.2.191 847061

[B5] Berli C. Lüscher J. Luszczynska A. Schwarzer R. Scholz U. (2018a). Couples’ daily self-regulation: the health action process approach at the dyadic level. *PLoS One* 13:e0205887. 10.1371/journal.pone.0205887 30372470 PMC6205589

[B6] Berli C. Schwaninger P. Scholz U. (2021). “We Feel Good”: daily support provision, health behavior, and well-being in romantic couples. *Front. Psychol.* 11:622492. 10.3389/fpsyg.2020.622492 33536986 PMC7848131

[B7] Berli C. Stadler G. Inauen J. Scholz U. (2016). Action control in dyads: a randomized controlled trial to promote physical activity in everyday life. *Soc. Sci. Med.* 163 89–97. 10.1016/j.socscimed.2016.07.003 27421075

[B8] Berli C. Stadler G. Shrout P. E. Bolger N. Scholz U. (2018b). Mediators of physical activity adherence: results from an action control intervention in couples. *Ann. Behav. Med.* 52 65–76. 10.1007/s12160-017-9923-z 28710666

[B9] Bernstein M. Sloutskis D. Kumanyika S. Sparti A. Schutz Y. Morabia A. (1998). Data-based approach for developing a physical activity frequency questionnaire. *Am. J. Epidemiol.* 147 147–154. 10.1093/oxfordjournals.aje.a009427 9457004

[B10] Blair S. N. Cheng Y. Holder S. (2001). Is physical activity or physical fitness more important in defining health benefits? *Med. Sci. Sports Exerc.* 33(Suppl. 6) S379–S399. 11427763 10.1097/00005768-200106001-00007

[B11] Bundesamt für Sport BASPO [Federal Office of Sports], Bundesamt für Gesundheit BAG, and Gesundheitsförderung Schweiz, Netzwerk Gesundheit und Bewegung Schweiz (2009). *Gesundheitswirksame Bewegung [Health-enhancing Physical Activity].* Magglingen: BASPO.

[B12] Carr R. M. Prestwich A. Kwasnicka D. Thøgersen-Ntoumani C. Gucciardi D. F. Quested E. (2019). Dyadic interventions to promote physical activity and reduce sedentary behaviour: systematic review and meta-analysis. *Health Psychol. Rev.* 13 91–109. 10.1080/17437199.2018.1532312 30284501

[B13] Choi L. Liu Z. Matthews C. E. Buchowski M. S. (2011). Validation of accelerometer wear and nonwear time classification algorithm. *Med. Sci. Sports Exerc* 43 357–364. 20581716 10.1249/MSS.0b013e3181ed61a3 PMC3184184

[B14] Christakis N. A. Fowler J. H. (2008). The collective dynamics of smoking in a large social network. *N. Engl. J. Med.* 358 2249–2258. 10.1056/NEJMsa0706154 18499567 PMC2822344

[B15] Cohen J. (1988). *Statistical Power Analysis for the Behavioural Sciences*, 2nd Edn. New York, NY: Academic Press.

[B16] Fanning J. Mullen S. P. McAuley E. (2012). Increasing physical activity with mobile devices: a meta-analysis. *J. Med. Internet Res.* 14:e161. 10.2196/jmir.2171 23171838 PMC3514847

[B17] Findorff M. J. Wyman J. F. Gross C. R. (2009). Predictors of long-term exercise adherence in a community-based sample of older women. *J. Womens Health* 18 1769–1776. 10.1089/jwh.2008.1265 19951210 PMC2828261

[B18] Franckowiak S. C. Dobrosielski D. A. Reilley S. M. Walston J. D. Andersen R. E. (2011). Maximal heart rate prediction in adults that are overweight or obese. *J. Strength Cond. Res.* 25 1407–1412. 10.1519/jsc.0b013e3181d682d2 21116203 PMC3081386

[B19] Gorin A. A. Wing R. R. Fava J. L. Jakicic J. M. Jeffery R. West D. S. (2008). Weight loss treatment influences untreated spouses and the home environment: evidence of a ripple effect. *Int. J. Obes.* 32 1678–1684. 10.1038/ijo.2008.150 18762804 PMC2730773

[B20] Haapala I. Barengo N. C. Biggs S. Surakka L. Manninen P. (2009). Weight loss by mobile phone: a 1-year effectiveness study. *Public Health Nutr.* 12 2382–2391. 10.1017/s1368980009005230 19323865

[B21] Hagobian T. A. Phelan S. Schaffner A. Brannen A. McHugh A. Ashby-Thompson M. (2019). Ripple effect of lifestyle interventions during pregnancy on untreated partners’ weight. *Obesity* 27 733–739. 10.1002/oby.22447 30957985 PMC6478509

[B22] Head K. J. Noar S. M. Iannarino N. T. Grant Harrington N. (2013). Efficacy of text messaging-based interventions for health promotion: a meta-analysis. *Soc. Sci. Med.* 97 41–48. 10.1016/j.socscimed.2013.08.003 24161087

[B23] Heesch K. C. Mâsse L. C. Dunn A. L. Frankowski R. F. Mullen P. D. (2003). Does adherence to a lifestyle physical activity intervention predict changes in physical activity? *J. Behav. Med.* 26 333–348. 12921007 10.1023/a:1024205011001

[B24] Heron K. E. Smyth J. M. (2010). Ecological momentary interventions: incorporating mobile technology into psychosocial and health behaviour treatments. *Br. J. Health Psychol.* 15 1–39. 10.1348/135910709x466063 19646331 PMC2800172

[B25] Homish G. Leonard K. E. (2008). Spousal influence on general health behaviors in a community sample. *Am. J. Health Behav.* 32 754–763. 10.5555/ajhb.2008.32.6.754 18442354

[B26] Jackson S. E. Steptoe A. Wardle J. (2015). The influence of partner’s behavior on health behavior change: the English longitudinal study of ageing. *JAMA Intern. Med.* 175:385. 10.1001/jamainternmed.2014.7554 25599511

[B27] Johnson D. Deterding S. Kuhn K.-A. Staneva A. Stoyanov S. Hides L. (2016). Gamification for health and wellbeing: a systematic review of the literature. *Internet Interv.* 6 89–106. 30135818 10.1016/j.invent.2016.10.002 PMC6096297

[B28] Keller J. Hohl D. H. Hosoya G. Heuse S. Scholz U. Luszczynska A. (2020). Long-term effects of a dyadic planning intervention with couples motivated to increase physical activity. *Psychol. Sport Exerc.* 49:101710. 10.1016/j.psychsport.2020.101710

[B29] King H. A. Jeffreys A. S. McVay M. A. Coffman C. J. Voils C. I. (2014). Spouse health behavior outcomes from a randomized controlled trial of a spouse-assisted lifestyle change intervention to improve patient low-density lipoprotein cholesterol. *J. Behav. Med.* 37 1102–1107. 10.1007/s10865-014-9559-4 24584818

[B30] Knäuper B. Carrière K. Frayn M. Ivanova E. Xu Z. Ames-Bull A. (2018). The effects of if-then plans on weight loss: results of the McGill CHIP healthy weight program randomized controlled trial: effects of if-then plans. *Obesity* 26 1285–1295. 10.1002/oby.22226 29956503

[B31] Knoll N. Hohl D. H. Keller J. Schuez N. Luszczynska A. Burkert S. (2017). Effects of dyadic planning on physical activity in couples: a randomized controlled trial. *Health Psychol.* 36 8–20. 10.1037/hea0000423 27642760

[B32] Lüscher J. Stadler G. Scholz U. (2018). A daily diary study of joint quit attempts by dual-smoker couples: the role of received and provided social support. *Nicotine Tob. Res.* 20 100–107. 10.1093/ntr/ntx079 28387852

[B33] Maeder U. Martin B. W. Schutz Y. Marti B. (2006). Validity of four short physical activity questionnaires in middle-aged persons. *Med. Sci. Sports Exerc* 38 1255–1266. 10.1249/01.mss.0000227310.18902.28 16826022

[B34] Marcolino M. S. Oliveira J. A. Q. D’Agostino M. Ribeiro A. L. Alkmim M. B. M. Novillo-Ortiz D. (2018). The impact of mHealth interventions: systematic review of systematic reviews. *JMIR Mhealth Uhealth* 6:e23. 10.2196/mhealth.8873 29343463 PMC5792697

[B35] Michie S. Richardson M. Johnston M. Abraham C. Francis J. Hardeman W. (2013). The behavior change technique taxonomy (v1) of 93 hierarchically clustered techniques: building an international consensus for the reporting of behavior change interventions. *Ann. Behav. Med.* 46 81–95. 10.1007/s12160-013-9486-6 23512568

[B36] Murray J. M. Brennan S. F. French D. P. Patterson C. C. Kee F. Hunter R. F. (2017). Effectiveness of physical activity interventions in achieving behaviour change maintenance in young and middle aged adults: a systematic review and meta-analysis. *Soc. Sci. Med.* 192 125–133. 10.1016/j.socscimed.2017.09.021 28965003

[B37] Pew Research Center (2021). *Mobile Fact Sheet [Internet].* Available online at: https://www.pewresearch.org/internet/fact-sheet/mobile/ (accessed Auguest 4, 2021).

[B38] Prestwich A. Conner M. Lawton R. Bailey W. Litman J. Molyneaux V. (2005). Individual and collaborative implementation intentions and the promotion of breast self-examination. *Psychol. Health* 20 743–760. 10.1080/14768320500183335

[B39] Prince S. A. Adamo K. B. Hamel M. Hardt J. Connor Gorber S. Tremblay M. (2008). A comparison of direct versus self-report measures for assessing physical activity in adults: a systematic review. *Int. J. Behav. Nutr. Phys. Act.* 5:56. 10.1186/1479-5868-5-56 18990237 PMC2588639

[B40] Richards E. A. Franks M. M. McDonough M. H. Porter K. (2018). ‘Let’s move:’ a systematic review of spouse-involved interventions to promote physical activity. *Int. J. Health Promot. Educ.* 56 51–67. 10.1080/14635240.2017.1415160

[B41] Sasaki J. E. John D. Freedson P. S. (2011). Validation and comparison of ActiGraph activity monitors. *J. Sci. Med. Sport* 14 411–416. 10.1016/j.jsams.2011.04.003 21616714

[B42] Schierberl Scherr A. E. McClure Brenchley K. J. Gorin A. A. (2013). Examining a ripple effect: do spouses’ behavior changes predict each other’s weight loss? *J. Obes.* 2013:297268. 10.1155/2013/297268 24083021 PMC3777131

[B43] Scholz U. Berli C. (2014). A dyadic action control trial in overweight and obese couples (DYACTIC). *BMC Public Health* 14:1321. 10.1186/1471-2458-14-1321 25540972 PMC4364646

[B44] Scholz U. Sniehotta F. F. (2006). Langzeiteffekte einer Planungs- und Handlungskontrollintervention auf die körperliche Aktivität von Herzpatienten nach der Rehabilitation. *Z. Gesundheitspsychol.* 14 73–81. 10.1026/0943-8149.14.2.73

[B45] Scholz U. Berli C. Lüscher J. Knoll N. (2020). “Dyadic behavior change interventions,” in *The Handbook of Behavior Change [Internet]*, 1st Edn, eds Hagger M. S. Cameron L. D. Hamilton K. Hankonen N. Lintunen T. (Cambridge University Press), 632–648. Available online at: https://www.cambridge.org/core/product/identifier/9781108677318#CN-bp-43/type/book_part (accessed Auguest 6, 2021).

[B46] Schwarzer R. (2008). Modeling health behavior change: how to predict and modify the adoption and maintenance of health behaviors. *Appl. Psychol.* 57 1–29. 10.1023/a:1013593819121

[B47] Sniehotta F. F. Nagy G. Scholz U. Schwarzer R. (2006). The role of action control in implementing intentions during the first weeks of behaviour change. *Br. J. Soc. Psychol.* 45 87–106. 10.1348/014466605X62460 16573874

[B48] Stephens J. Allen J. (2013). Mobile phone interventions to increase physical activity and reduce weight: a systematic review. *J. Cardiovasc. Nurs.* 28 320–329. 10.1097/JCN.0b013e318250a3e7 22635061 PMC3681804

[B49] Stephenson A. McDonough S. M. Murphy M. H. Nugent C. D. Mair J. L. (2017). Using computer, mobile and wearable technology enhanced interventions to reduce sedentary behaviour: a systematic review and meta-analysis. *Int. J. Behav. Nutr. Phys. Act.* 14:105. 10.1186/s12966-017-0561-4 28800736 PMC5553917

[B50] Telford R. Minikin B. Hooper L. A. Hahn A. G. (1989). A simple method for the assessment of general fitness: the tri-level profile. *Aust. J. Sci. Med. Sport* 21 6–9.

[B51] Tutunchi H. Ebrahimi-Mameghani M. Ostadrahimi A. Asghari-Jafarabadi M. (2020). What are the optimal cut-off points of anthropometric indices for prediction of overweight and obesity? Predictive validity of waist circumference, waist-to-hip and waist-to-height ratios. *Health Promot. Perspect.* 10 142–147. 10.34172/hpp.2020.23 32296627 PMC7146042

[B52] Wallman K. Campbell L. (2007). Test–retest reliability of the Aerobic Power Index submaximal exercise test in an obese population. *J. Sci. Med. Sport* 10 141–146. 10.1016/j.jsams.2006.05.024 16844410

[B53] Wallman K. Goodman C. Morton A. Grove R. Dawson B. (2003). Test-retest reliability of the aerobic power index component of the tri-level fitness profile in a sedentary population. *J. Sci. Med. Sport* 6 443–454. 10.1016/s1440-2440(03)80270-0 14723394

[B54] Wang J. B. Cadmus-Bertram L. A. Natarajan L. White M. M. Madanat H. Nichols J. F. (2015). Wearable sensor/device (Fitbit one) and SMS text-messaging prompts to increase physical activity in overweight and obese adults: a randomized controlled trial. *Telemed. eHealth* 21 782–792. 10.1089/tmj.2014.0176 26431257 PMC4842945

[B55] WHO (2008). *Waist Circumference and Waist-Hip Ratio: Report of a WHO Expert Consultation [Internet].* Geneva: World Health Organization.

[B56] WHO (2020). *Physical activity [Internet].* Available online at: https://www.who.int/news-room/fact-sheets/detail/physical-activity (accessed July 26, 2021).

[B57] WHO (2021). *Obesity and overweight [Internet].* Geneva: World Health Organization

[B58] Wilson S. E. (2002). The health capital of families: an investigation of the inter-spousal correlation in health status. *Soc. Sci. Med.* 55 1157–1172. 10.1016/s0277-9536(01)00253-2 12365528

[B59] Winters-Stone K. M. Lyons K. S. Dobek J. Dieckmann N. F. Bennett J. A. Nail L. (2016). Benefits of partnered strength training for prostate cancer survivors and spouses: results from a randomized controlled trial of the Exercising Together project. *J. Cancer Surviv.* 10 633–644. 10.1007/s11764-015-0509-0 26715587

[B60] Yang Q. Van Stee S. K. (2019). The comparative effectiveness of mobile phone interventions in improving health outcomes: meta-analytic review. *JMIR Mhealth Uhealth* 7:e11244. 10.2196/11244 30942695 PMC6468337

[B61] Yates B. C. Norman J. Meza J. Krogstrand K. S. Harrington S. Shurmur S. (2015). Effects of partners together in health intervention on physical activity and healthy eating behaviors: a pilot study. *J. Cardiovasc. Nurs.* 30 109–120. 10.1097/jcn.0000000000000127 24434826 PMC4098014

