# Supplementary material for: Long-Term and Transfer Effects of an Action Control Intervention in Overweight Couples: A Randomized Controlled Trial Using Text Messages
Source: Front Psychol. 2021 Nov 24;12:754488. doi: 10.3389/fpsyg.2021.754488 (PMC8651541; doi:10.3389/fpsyg.2021.754488)
Supplement: Supplementary file 1 [file Data_Sheet_1.docx]

SUPPLEMENTAL MATERIAL A

## Self-reported physical activity questionnaire

***PLEASE NOTE:***

*The following physical activity questionnaire was adapted from the physical activity frequency questionnaire PAFQ from Bernstein and colleagues (1998), and from its advanced version OIMQ from Mäder and colleagues (2006). The item about being active together with the partner was internally developed and added to the questionnaire. Please note that the following questionnaire represents a simple English translation from the German original for transparency.*

**REFERENCES:**

Bernstein, M., Sloutskis, D., Kumanyika, S., Sparti, A., Schutz, Y. & Morabia, A. (1998). Data-based approach for developing a physical activity frequency questionnaire. *American Journal of Epidemiology, 147*, 147-154.

Maeder, U., Martin, B. W., Schutz, Y. & Marti, B. (2006). Validity of four short physical activity questionnaires in middle-aged persons. *Medicine & Science in Sports & Exercise, 38,* 1255-1266.

For each of the activities listed, please indicate by marking with a cross, how often you performed the activity during the **last seven days.** Please mark the appropriate box with a cross. If you did not perform the activity, please mark «0» and continue with the following activity.

For all activities performed please indicate the **average duration of physical activity *per day*** (based on the days you were active) as well as how many minutes per day on average you were active **with your partner** (if you performed the activity without your partner please mark “no”).

At the end of the questionnaire, you will have the possibility to add activities that are not listed.

1. **Domestic and Garden Activities**

| **Activities** | **Number of days** | | | | | | | | **Average duration per day** | **Active together with your partner?** |
| --- | --- | --- | --- | --- | --- | --- | --- | --- | --- | --- |
|  | 0 | 1 | 2 | 3 | 4 | 5 | 6 | 7 |  |  |
| Sitting still  (e.g. reading, watching tv, cinema, card game, writing, computer) |  |  |  |  |  |  |  |  | _______ min. | ❑ yes, namely: _______ min.  ❑ no |
| Cooking, washing up, ironing, sewing |  |  |  |  |  |  |  |  | _______ min. | ❑ yes, namely: _______ min.  ❑ no |
| Laundry, vacuuming |  |  |  |  |  |  |  |  | _______ min. | ❑ yes, namely: _______ min.  ❑ no |
| Childcare with carrying |  |  |  |  |  |  |  |  | _______ min. | ❑ yes, namely: _______ min.  ❑ no |
| Gardening work (e.g. weeding, raking, hedge trimming, lawn mowing) |  |  |  |  |  |  |  |  | _______ min. | ❑ yes, namely: _______ min.  ❑ no |

1. **Active Transportation** (e.g. going to work, shopping, going out)

| **Activities** | **Number of days** | | | | | | | | **Average duration per day** | **Active together with your partner?** |
| --- | --- | --- | --- | --- | --- | --- | --- | --- | --- | --- |
|  | 0 | 1 | 2 | 3 | 4 | 5 | 6 | 7 |  |  |
| Walking normally |  |  |  |  |  |  |  |  | _______ min. | ❑ yes, namely: _______ min.  ❑ no |
| Walking quickly or uphill |  |  |  |  |  |  |  |  | _______ min. | ❑ yes, namely: _______ min.  ❑ no |
| Walking while carrying heavy baggage |  |  |  |  |  |  |  |  | _______ min. | ❑ yes, namely: _______ min.  ❑ no |
| Climbing up stairs |  |  |  |  |  |  |  |  | Number of storeys:  _______ | ❑ yes, namely: _______  ❑ no |
| Cycling slowly  (about 9 km/h) |  |  |  |  |  |  |  |  | _______ min. | ❑ yes, namely: _______ min.  ❑ no |
| Cycling fast  (about 15 km/h) |  |  |  |  |  |  |  |  | _______ min. | ❑ yes, namely: _______ min.  ❑ no |
| Using public transport |  |  |  |  |  |  |  |  | _______ min. | ❑ yes, namely: _______ min.  ❑ no |
| Driving a car or a motorcycle |  |  |  |  |  |  |  |  | _______ min. | ❑ yes, namely: _______ min.  ❑ no |

*Note: The item using public transport has been added from the OIMQ (Mäder et al., 2006)*

1. **Leisure-Time Activities and Sport**

| **Activities** | **Number of days** | | | | | | | | **Average duration per day** | **Active together with your partner?** |
| --- | --- | --- | --- | --- | --- | --- | --- | --- | --- | --- |
|  | 0 | 1 | 2 | 3 | 4 | 5 | 6 | 7 |  |  |
| Cycling (exercise)  (if not for transportation) |  |  |  |  |  |  |  |  | _______ min. | ❑ yes, namely: _______ min.  ❑ no |
| Going for a walk |  |  |  |  |  |  |  |  | _______ min. | ❑ yes, namely: _______ min.  ❑ no |
| Hiking |  |  |  |  |  |  |  |  | _______ min. | ❑ yes, namely: _______ min.  ❑ no |
| Athletic Walking |  |  |  |  |  |  |  |  | _______ min. | ❑ yes, namely: _______ min.  ❑ no |
| Running, Jogging |  |  |  |  |  |  |  |  | _______ min. | ❑ yes, namely: _______ min.  ❑ no |
| Team sports  (e.g. soccer, basketball, handball) |  |  |  |  |  |  |  |  | _______ min. | ❑ yes, namely: _______ min.  ❑ no |
| Gymnastics |  |  |  |  |  |  |  |  | _______ min. | ❑ yes, namely: _______ min.  ❑ no |
| Weightlifting, training on equipment |  |  |  |  |  |  |  |  | _______ min. | ❑ yes, namely: _______ min.  ❑ no |
| Fitness gymnastics (z.B. aerobics, pilates, yoga) |  |  |  |  |  |  |  |  | _______ min. | ❑ yes, namely: _______ min.  ❑ no |
| Dancing (e.g. ballet, rock’n roll) |  |  |  |  |  |  |  |  | _______ min. | ❑ yes, namely: _______ min.  ❑ no |
| Swimming |  |  |  |  |  |  |  |  | _______ min. | ❑ yes, namely: _______ min.  ❑ no |
| Alpine skiing |  |  |  |  |  |  |  |  | _______ min. | ❑ yes, namely: _______ min.  ❑ no |
| Cross-country skiing |  |  |  |  |  |  |  |  | _______ min. | ❑ yes, namely: _______ min.  ❑ no |
| Tennis (single), badminton |  |  |  |  |  |  |  |  | _______ min. | ❑ yes, namely: _______ min.  ❑ no |
| Tennis (doubles) |  |  |  |  |  |  |  |  | _______ min. | ❑ yes, namely: _______ min.  ❑ no |
| Squash |  |  |  |  |  |  |  |  | _______ min. | ❑ yes, namely: _______ min.  ❑ no |
| Golf |  |  |  |  |  |  |  |  | _______ min. | ❑ yes, namely: _______ min.  ❑ no |
| Judo, karate |  |  |  |  |  |  |  |  | _______ min. | ❑ yes, namely: _______ min.  ❑ no |
| Ice or roller skating |  |  |  |  |  |  |  |  | _______ min. | ❑ yes, namely: _______ min.  ❑ no |

1. **Additional activities (Sport and other)**

In the table below please enter physical activities you performed that were *not* listed earlier.

| **Activities** | **Number of days** | | | | | | | | **Average duration per day** | **Active together with your partner?** |
| --- | --- | --- | --- | --- | --- | --- | --- | --- | --- | --- |
|  | 0 | 1 | 2 | 3 | 4 | 5 | 6 | 7 |  |  |
| 1. |  |  |  |  |  |  |  |  | _______ min. | ❑ yes, namely: _______ min.  ❑ no |
| 2. |  |  |  |  |  |  |  |  | _______ min. | ❑ yes, namely: _______ min.  ❑ no |
| 3. |  |  |  |  |  |  |  |  | _______ min. | ❑ yes, namely: _______ min.  ❑ no |
