# Supplementary material for: Long-Term and Transfer Effects of an Action Control Intervention in Overweight Couples: A Randomized Controlled Trial Using Text Messages
Source: Front Psychol. 2021 Nov 24;12:754488. doi: 10.3389/fpsyg.2021.754488 (PMC8651541; doi:10.3389/fpsyg.2021.754488)
Supplement: Supplementary file 2 [file Table_1.docx]

SUPPLEMENTAL MATERIAL B

Table S1. *Descriptive statistics of primary and secondary outcomes for target persons and partners at baseline (T1), one-month (T2) and 6-month (T3) follow-up in the dyadic vs. individual action control intervention groups.*

|  |  | Target persons | |  | Partners | |  |
| --- | --- | --- | --- | --- | --- | --- | --- |
|  |  | Dyadic IG  (*n* = 30) | Individual IG  (*n* = 30) |  | Dyadic IG  (*n* = 30) | Individual IG  (*n* = 30) |  |
|  |  | *M (SD)* | *M (SD)* | *t* | *M (SD)* | *M (SD)* | *t* |
| Self-reported MVPA (min/day) | T1 | 188.55 (241.22) | 125.57 (141.57) | -1.23 | 169.12 (185.36) | 201.31 (182.86) | 0.68 |
|  | T2 | 197.49 (210.49) | 148.20 (132.89) | -1.08 | 154.17 (120.70) | 192.59 (224.68) | 0.81 |
|  | T3 | 150.25 (143.28) | 136.79 (116.70) | -0.37 | 152.04 (164.12) | 179.84 (170.81) | 0.57 |
| Objective MVPA (min/day) | T3 | 48.99 (31.02) | 45.17 (24.60) | -0.48 | 45.25 (17.54) | 46.19 (24.98) | 0.15 |
| Objective MVPA adherence | T3 | 0.28 (0.29) | 0.22 (0.22) | -0.87 | 0.26 (0.22) | 0.16 (0.16) | -1.72^†^ |
| BMI | T1 | 31.27 (5.55) | 32.63 (5.55) | 0.94 | 33.58 (5.18) | 31.62 (4.04) | -1.64 |
|  | T2 | 31.23 (5.06) | 32.54 (5.51) | 0.89 | 33.05 (5.69) | 31.54 (4.24) | -1.15 |
|  | T3 | 30.94 (4.23) | 32.47 (5.82) | 0.99 | 33.56 (4.99) | 31.54 (3.89) | -1.61 |
| Waist-to-hip Ratio | T1 | 0.92 (0.07) | 0.92 (0.08) | 0.01 | 0.92 (0.10) | 0.92 (0.08) | -0.12 |
|  | T2 | 0.90 (0.07) | 0.91 (0.08) | 0.57 | 0.92 (0.10) | 0.91 (0.09) | -0.37 |
|  | T3 | 0.92 (0.06) | 0.91 (0.08) | -0.75 | 0.90 (0.11) | 0.93 (0.08) | 0.93 |
| Physical fitness (Wkg^-1^) | T1 | 1.40 (0.52) | 1.40 (0.32) | -0.01 | - | - |  |
|  | T3 | 1.48 (0.52) | 1.41 (0.28) | -0.51 | - | - |  |

Note. T1: N = 121; T2: N=115; T3: N=99; M= Mean, SD = Standard deviation. ^†^*p* < .10; for all other t’s p > .05.
